# Subthreshold Vibrotactile Noise Stimulation Immediately Improves Manual Dexterity in a Child With Developmental Coordination Disorder: A Single-Case Study

**DOI:** 10.3389/fneur.2019.00717

**Published:** 2019-07-02

**Authors:** Satoshi Nobusako, Michihiro Osumi, Atsushi Matsuo, Emi Furukawa, Takaki Maeda, Sotaro Shimada, Akio Nakai, Shu Morioka

**Affiliations:** ^1^Neurorehabilitation Research Center, Kio University, Koryo, Japan; ^2^Graduate School of Health Science, Kio University, Koryo, Japan; ^3^Department of Physical Therapy, Faculty of Health Sciences, Kio University, Koryo, Japan; ^4^Department of Neuropsychiatry, Keio University School of Medicine, Tokyo, Japan; ^5^Department of Electronics and Bioinformatics School of Science and Technology, Meiji University, Kawasaki, Japan; ^6^Graduate School of Clinical Education & The Center for the Study of Child Development, Institute for Education, Mukogawa Women's University, Nishinomiya, Japan

**Keywords:** delayed visual feedback detection task, DCD, manual dexterity, sensory-dependence, stochastic resonance (SR), temporal order judgment (TOJ) task, vibrotactile noise stimulation, visuo-motor temporal integration

## Abstract

Developmental coordination disorder (DCD) is the most common childhood movement disorder. It is characterized by clumsiness of fine and gross motor skills in developing children. Children with DCD have low ability to effectively use tactile information for movements, instead relying on visual information. In addition, children with DCD have deficits in visuo-motor temporal integration, which is important in motor control. These traits subsequently lead to clumsiness of movements. Conversely, however, imperceptible vibrotactile noise stimulation (at 60%-intensity of the sensory threshold) to the wrist provides stochastic resonance (SR) phenomenon to the body, improving the sensory and motor systems. However, the effects of SR have not yet been validated in children with DCD. Thus, we conducted a single case study of a 10-year-old boy with a diagnosis of DCD to investigate the effect of SR on visual dependence, visuo-motor temporal integration, and manual dexterity. SR was provided by vibrotactile noise stimulation (at an intensity of 60% of the sensory threshold) to the wrist. Changes in manual dexterity (during the SR on- and off-conditions) were measured using the manual dexterity test of the Movement Assessment Battery for Children-2nd edition. The point of subjective equality measured by visual or tactile temporal order judgment task served as a quantitative indicator reflecting specific sensory dependence. The delay detection threshold and steepness of delay detection probability curve, which were measured using the delayed visual feedback detection task, were used as quantitative indicators of visuo-motor temporal integration. The results demonstrated alleviated visual dependence and improved visuo-motor temporal integration during the SR on-conditions rather than the SR off-conditions. Most importantly, manual dexterity during the SR on-conditions was significantly improved compared to that during the SR off-conditions. Thus, the present results highlighted that SR could contribute to improving poor movement in children with DCD. However, since this was a single case study, a future interventional study with a large sample size is needed to determine the effectiveness of SR for children with DCD.

## Introduction

Developmental coordination disorder (DCD), which is characterized by clumsiness in fine and gross motor skills, affects ~6% of school-aged children, making it the most common childhood movement disorder (1–4). Children with DCD have lower ability to effectively use tactile information for movement, instead relying on visual information. Several studies have shown that increased visual dependence in children with DCD has a negative impact on the success of motor tasks (5–12). In addition, children with DCD have deficits in sensory-motor integration. Many previous studies have shown that deficits in sensory-motor integration have been linked to clumsy movements (13–23). In the current case study, we focused on the manual dexterity of a child with DCD. Recent research and review articles have shown that the clumsiness of manual dexterity in individuals with DCD is associated with the reduced activity of the premotor cortex and inferior parietal lobe, i.e., the frontal-parietal network (24–27). Therefore, it is suggested that the effective activation of the frontal-parietal network may improve manual dexterity in DCD.

On the other hand, sensory subthreshold mechanical noise stimulation to the body is known to improve the sensory-motor system. This improvement is related to stochastic resonance (SR), a phenomenon described as a “noise benefit” to various sensory and motor systems (28). SR application has been shown to improve the sensitivity of the visual (29), auditory (30), vestibular (31), and tactile (32–36) sensory systems. In addition, previous studies have demonstrated the immediate improvement in posture balance, walking, and hand movements following the application of SR (32, 34, 35, 37–41). These improvements were observed not only in healthy participants but also in older adults and patients with diabetes, stroke, and Parkinson's disease, and children with cerebral palsy (32–34, 38–40, 42, 43).

Vibrotactile noise stimulation to the wrist at an intensity of 60% of the sensory threshold generates SR phenomena in the hand, which in turn improve the tactile sensitivity of the fingertips and manual dexterity (34, 36, 40, 41). This improvement is thought to be caused by SR acting on the peripheral and central nervous systems. Vibrotactile noise stimulation can also enhance sensory sensitivity by directly stimulating peripheral sensory receptors (35). In addition, vibrotactile noise stimulation increases cortical and spinal neuronal activity (44–46). Importantly, this increase is not only limited to the sensorimotor cortex but also extends to the premotor and posterior parietal cortices (46, 47), which are important for tactile sensitivity (46), visuo-motor temporal integration (48), and manual dexterity (24–27). Further, studies have shown that vibrotactile noise increases the synchronization of neuronal firing between the spinal cord and sensorimotor cortex and between different brain areas (44, 45, 49–51). This increased neural synchronization can facilitate neural communication for perception between spinal and cortical levels (49, 52). Therefore, the application of SR to children with DCD may improve the clumsiness of movements; however, this has not yet been verified.

In the current case study, we hypothesized that the application of vibrotactile noise stimulation to the wrist with an intensity of 60% of the sensory threshold in children with DCD could reduce visual dependence by enhancing tactile sensitivity, promoting visuo-motor temporal integration, and improving poor manual dexterity. To verify this hypothesis, we applied SR to a 10-year-old boy with DCD and measured changes in manual dexterity, sensory dependence, and visuo-motor temporal integration.

## Materials and Methods

### Case

A 10-year-old boy was examined by a neuro-pediatrician specialist 1 year before the current study and was diagnosed with DCD according to the Diagnostic and Statistical Manual of Mental Disorders 5th edition (DSM-5) (1). The boy had no other diagnosis of a general medical condition (e.g., cerebral palsy, hemiplegia, and muscular dystrophy), other developmental disorder (e.g., autism spectrum disorder, attention deficit hyperactivity disorder, and learning disorder), or intellectual disability. The experimental procedures were approved by the local ethics committee of the Graduate School and Faculty of Health Sciences at Kio University (approval number: 15–33). There were no foreseeable risks to the patient. No personal identification information was collected. We explained the study to the patient and his parents. The patient and his parents provided written informed consent for participation in this study and publication of this study. The procedures complied with the ethical standards of the 1964 Declaration of Helsinki regarding the treatment of human participants in research.

The boy's motor function and depression tendency were evaluated using the Movement Assessment Battery for Children-2nd edition (M-ABC-2) (53) and Depression Self-Rating Scale for Children (DSRS-C) (54), respectively, 1 day before carrying out the current study (Table 1).

**Table 1:** Results of tests conducted on the day before the current study.

Sex			Male
Age (years)			10
Preferred hand			Right
M-ABC-2	Manual dexterity component score		32
	Manual dexterity standard score		11
	Manual dexterity percentile		63
	Aiming & catching component score		12
	Aiming & catching standard score		5
	Aiming & catching percentile		5
	Balance component score		16
	Balance standard score		5
	Balance percentile		5
	Total test score		60
	Standard score		6
	Percentile rank		9
DCDQ	Control during movement		14
	Fine motor and Handwriting		8
	General coordination		7
	Total score		29
SCQ			9
ADHD-RS	Inattention	Score	11
		Percentile	88
	Hyperactivity-Impulsivity	Score	5
		Percentile	84
	Total	Score	16
		Percentile	87
DSRS-C			3
Temporal order judgment task (Sensory dependence)	PSE (ms)		−24.77
Delayed visual feedback detection task (Visuo-motor temporal integration)	DDT (ms)		275.7
	Steepness		0.02673

*M-ABC2, Movement Assessment Battery for Children-2nd Edition; DCDQ, Developmental Coordination Disorder Questionnaire; SCQ, Social Communication Questionnaire; ADHD-RS, Attention-Deficit Hyperactivity Disorder Rating Scale; DSRS-C, Depression Self-Rating Scale for Children; PSE, point of subjective equality; DDT, delay detection threshold: Steepness, steepness of the probability curve for delay detection*.

The patient's parents also completed the Japanese version of the Developmental Coordination Disorder Questionnaire (DCDQ) (55), Social Communication Questionnaire (SCQ) (56), and Attention-Deficit Hyperactivity Disorder Rating Scale (ADHD-RS) (57), 1 day prior to conducting the current study to evaluate the patient's motor function (55), autism spectrum disorder (ASD) traits (56), and ADHD traits (57), respectively (Table 1). In addition, the patient performed temporal order judgment (TOJ) and delayed visual feedback detection tasks to evaluate sensory-dependent tendency and visuo-motor temporal integration, respectively (Table 1).

M-ABC-2 is an international standard evaluation battery for evaluating DCD diagnostic criteria A of DSM-5 (53) and DCDQ is a parent's rating scale for evaluating DCD diagnostic criterion B (55). In order to satisfy the DCD diagnostic criteria A of DSM-5, it is recommended that it be less than the 16th percentile as measured by M-ABC-2. The Japanese version of M-ABC-2, which is now being developed (58), has not been standardized. Thus, the original UK data were used when raw scores were converted to a standardized score or percentile. In order to satisfy the DCD diagnostic criterion B of DSM-5, it is recommended that it is 57 points or less as measured by DCDQ. The patient was in the 9th percentile of the M-ABC-2 and had 29 points according to the DCDQ; thus, he was diagnosed with DCD. The score of SCQ was nine points, ASD traits were low. The percentile of the ADHD-RS was 88th percentile for the inattention item, 84th percentile for the hyperactivity-impulsivity item, and 87th percentile for the total. The score of DSRS-C was three points, and no depression tendency was observed. He was not receiving any ongoing habilitation or medication therapy at the time of participating in the current study.

### Procedures

**Figure 1:**
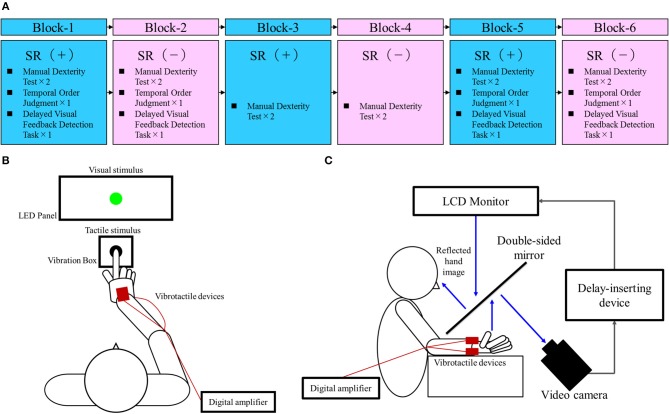
Block design of the experimental protocol and experimental tasks. **(A)** Block design of the experimental protocol. Blue squares, stochastic resonance (SR) on-condition (+); Pink squares, SR off-condition (–). **(B)** Temporal order judgment task. A visuo-tactile temporal order judgment (TOJ) device (Keio method, UT-0021, Medical Try System, Tokyo, Japan) was used for the present task. This device included an LED panel (UT-0021-2, Medical Try System, Tokyo, Japan) and vibration box (UT-0021-1, Medical Try System, Tokyo, Japan), which provided the visual and tactile stimuli, respectively. The child put the index finger of his right hand in the hole of the vibration box and contacted the vibrotactile stimulator. Therefore, the case could not observe the tactile stimulus. The child was requested to watch the LED panel. The TOJ task device was to set conditions for the synchronized presentation of the visual and tactile stimuli, and the presentation of the visual stimulus at 50 and 100 ms earlier than the tactile stimulus (or vice versa). In addition, the setup included a blackout curtain so that the child would not be able to see outside the experimental chamber. **(C)** Delayed visual feedback detection task. The child's right hand was placed under a two-way mirror so that he was unable to see his right hand directly. The image of the hand, which was reflected in the two-sided mirror, was filmed with a video camera (FDR-AXP35, Sony, Tokyo, Japan). The movie of the photographed hand was further reflected from an installed monitor (LMD-A240, Sony, Tokyo, Japan) onto the two-sided mirror via a video delay-inserting device (EDS-3306, FOR-A YEM ELETEX, Tokyo, Japan). Thus, the child observed the delayed image of his right hand reflected in the mirror at the position where his right hand would be. In addition, the setup included a blackout curtain so that the child would not be able to see outside the experimental chamber. The intrinsic delay of the visual feedback in this experimental setting was 33.7 ms as measured by a time lag check device (EDD-5200, FOR-A YEM ELETEX, Tokyo, Japan).

Figure 1A outlines the block design of the experimental protocol. There were three blocks each of the SR on-condition and SR off-condition (in order of SR on-off-on-off-on-off), with six blocks in total. Blocks 1, 3, and 5 were the SR on-condition, while blocks 2, 4, and 6 were the SR off-condition. This order was designed to offset the learning effects of repeating the test. Each block contained two manual dexterity tests, with a total of 12 manual dexterity tests performed throughout the study. That is, a total of 12 manual dexterity tests were performed six times each under the SR on-condition (Blocks 1, 3, and 5) and SR-off condition (Blocks 2, 4, and 6). The temporal order judgment task and delayed visual feedback detection task was administered once each during Block 1 and 5 (first and last SR on-condition) and Block 2 and 6 (first and last SR off-condition), respectively. This design was intended to reduce the burden on the patient.

### Stochastic Resonance

Vibrotactile noise was applied using four compact devices (vertical, 10 mm; width, 18 mm; height, 2 mm; Vibration Actuator Sprinter α; Nidec Seimitsu, Nagano, Japan) attached to the volar and dorsal areas of the child's right and left wrists, respectively, using contact tape (i.e., two devices on the right wrist and two devices on the left wrist). The resonance frequency of the device was 170 ± 10 Hz (average ± SD); low-pass filters at 500 Hz were used as per previous studies (34, 36, 40, 41, 46). A digital amplifier (FX Audio D802; North Flat Japan, Osaka, Japan) was used to output the white noise signals to the SR device (a vibrotactile noise device). Consistent with previous protocols (34, 36, 40, 41, 46), we attached the device to the wrist to minimize manual interruption while affecting the tactile sensation of the fingers. The intensity of the vibrotactile noise was set to 60% of the sensory threshold at the start of the test—the optimum level to affect the sensory system (33, 34, 36, 40, 41, 46). The sensory thresholds of the vibrotactile noise were measured immediately before starting each of the six blocks, irrespective of whether it was an SR on- or off-condition. The vibrotactile noise device was attached at all times during testing and was turned on or off at the beginning of each block according to the SR on-/off-conditions used. The patient was blinded to the condition as he could not feel the noise vibrations.

### Manual Dexterity Test

The manual dexterity test of the M-ABC-2 is a standardized, age-adjusted test to evaluate the DCD diagnostic criteria A of DSM-5 (53). Since the patient was 10 years old, we conducted three sub-tests of age band-2 to evaluate manual dexterity; placing pegs test (Manual dexterity 1), threading lace test (Manual dexterity 2), and drawing trail II test (Manual dexterity 3). The patient was wearing vibrotactile noise devices on the right and left wrists during this test. This test was conducted twice in each block (Blocks 1, 3, and 5 as the SR on-conditions, and Blocks 2, 4, and 6 as the SR off-conditions), with a total of 12 tests conducted throughout the whole experiment. The component score, standard score, and percentile were then calculated from the obtained raw scores. An increase in the component score, standard score, and percentile represented an improvement in manual dexterity. This assessment was administered by a specifically trained, certified physical therapist.

### Temporal Order Judgment Task

Sensory dependence was measured using the temporal order judgment (TOJ) task (59–63) (Figure 1B), where two stimuli (visual-flashes; tactile-vibrations) were presented in several stimulus onset asynchronies (SOA). The child was then required to determine which stimulus (visual or tactile) was presented first. This visuo-tactile TOJ task was carried out using a TOJ task device (Keio method, UT-0021, Medical Try System, Tokyo, Japan). Visual stimulation was elicited by a green LED in an LED panel (UT-0021-2, Medical Try System, Tokyo, Japan). The luminance of the visual stimulus was 40 cd/m^2^ and the duration of visual stimulation was 1 ms. A 1-ms tactile stimulus (converted to vibration by pneumatic pressure) was administered to the right index finger controlled by a 1-V signal from the vibration box (UT-0021-1, Medical Try System, Tokyo, Japan). The stimulation condition included the following five conditions: (at −100, −50, 0, 50, 100 ms), i.e., four conditions where visual or tactile stimulation was administered 50 or 100 ms earlier than the other (i.e., tactile first, −100, −50 ms; visual first, 50, 100 ms), and a synchronous condition of visual and tactile stimulation (0 ms). During each block, the five stimulation conditions were considered a set and the child performed five sets; the trial order was randomized. Therefore, the child completed 100 trials with four blocks. This task was performed with the child's right hand attached to the vibrotactile noise devices.

Before starting the TOJ task, simple stimulus tests were used to confirm that the patient had no problems with vision and touch. First, the visual stimulus was not presented, and only the tactile stimulus was given five times to determine if there was a problem with tactile input. Subsequently, no tactile stimulus was presented, and only five visual stimuli were given to confirm whether there was a problem with visual input. The visual and tactile stimuli used for confirmation were the same as the stimuli used in the TOJ task. These simple stimulus tests confirmed that the patient was able to perceive tactile and visual stimuli.

For the TOJ task, the “visual first” response probability for each several SOA conditions (−100, −50, 0, 50, 100) was then calculated. Logistic curves were fitted to the “visual first” response probability in the TOJ task on the basis of following formula (23, 64, 65).

$$\begin{array}{l} P\left(t\right)\;=\;\frac{1}{1+\exp\left(\;-a\left(t-t_{PSE}\right)\right)} \end{array}$$

where t is the SOA; P(t) is the probability of “visual first” response; a indicates the steepness of the fitted curve; and tPSE indicates the observer's point of subjective equality (PSE), which demonstrates the SOA where “visual first” and “tactile first” judgment probabilities are equal (50%). Data were fitted using a non-linear least squares algorithm in MATLAB R2014b (MathWorks, MA, USA). Further, the PSE of each of the four blocks including the SR on-conditions (Blocks 1 and 5) and SR off-conditions (Blocks 2 and 6) was calculated. The PSE was a sensory-dependent quantitative indicator, where a large negative PSE value showed visual dependence and a large positive PSE value showed tactile dependence. Therefore, a PSE value approaching 0 ms demonstrated no biased sensory dependence. As baseline data, this TOJ task was also conducted 1 day before the current study, with the SR devices not attached (Table 1).

### Delayed Visual Feedback Detection Task

The delayed visual feedback detection task was carried out using the same setting as previous studies (23, 65–67) (Figure 1C). The child performed the task with his right hand, which was connected to the vibrotactile noise devices. After the experimenter had informed him orally that the trial had started, the child opened and closed his right hand once in a continuous and smooth manner, according to his own volition. The self-generated movements were observed under the following 18 delay conditions using a video delay-inserting device: 33, 67, 100, 133, 167, 200, 233, 267, 300, 333, 367, 400, 433, 467, 500, 533, 567, and 600 ms. The child had to determine if the visual feedback was synchronous or asynchronous relative to the movement of his right hand. Immediately following the trial, the child had to state orally if the visual feedback was “delayed” or “not delayed” by using the forced-choice method. In each block, all 18 delay conditions were treated as one set; their presentation order was randomized. Four sets were performed in total. The task was carried out once during each of the first and last SR on-block (Blocks 1 and 5) and SR off-block (Blocks 2 and 6), respectively; a total of four tests were carried out. Therefore, the child completed a total of 72 randomized trials with 18 delay conditions per set of four per block. Since there were four blocks in total, with or without SR, a total of 288 randomized tests were completed. Before the task, we confirmed that the patient could distinguish between a minimum delay of 33 ms and a maximum delay of 600 ms. That is, before the task, he reported “not delayed” for the minimum delay of 33 ms and reported “delayed” for the maximum delay of 600 ms.

The delay detection threshold (DDT) and steepness of the probability curve for delay detection, which will be referred to herein as “steepness,” were determined using this task. Shortened DDT and/or increased steepness represented high visuomotor temporal integration, while prolonged DDT and/or decreased steepness represented poor visuomotor temporal integration. A logistic curve was fitted to the child's response on the visual feedback delay detection task, using the following formula (23, 64, 65):

$$\begin{array}{l} P\left(t\right)\;=\;\frac{1}{1+\exp\left(\;-a\left(t-DDT\right)\right)} \end{array}$$

where t was the visual feedback delay length (independent variable); P(t) was the probability of delay detection (observed value); a was the steepness of the fitted curve; and DDT was the observer's DDT representing the delay length at which the probability of delay detection was 50%. The curve was fitted using a non-linear least squares method (a trust-region algorithm) with MATLAB R2014b (MathWorks, Inc., Natick, MA, USA) to estimate a and DDT. DDT and the steepness of each of the four blocks including the SR on-conditions (Blocks 1 and 5) and SR off-conditions (Blocks 2 and 6) were calculated. As baseline data, this task was also conducted 1 day before the current study, with the SR devices not attached (Table 1).

### Statistical Analysis

The results of the manual dexterity test under the SR on-conditions (a total of six test results of twice each in Blocks 1, 3, and 5) and the results of the manual dexterity test under the SR off-conditions (a total of six test results of twice each in Blocks 2, 4, and 6) were compared. Manual dexterity test scores (component score, standard score, percentile) were compared using the Wilcoxon signed-rank test, since they were not normally distributed by the Shapiro-Wilk test. In addition, the effect size was calculated (68). The significance level was set at *P* < 0.05. All statistical analyses were performed using SPSS ver. 24 (SPSS, Chicago, IL, USA).

## Results

**Figure 2:**
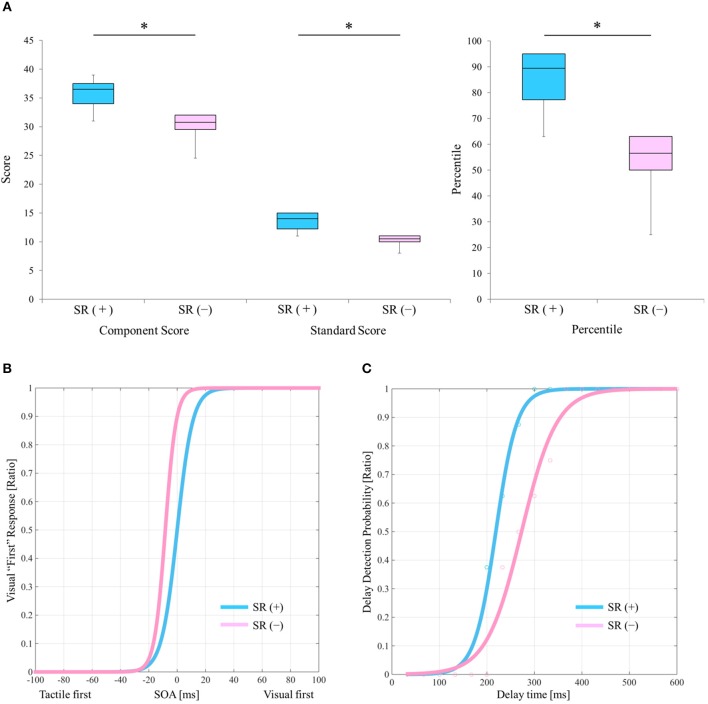
Results of the manual dexterity test and experimental tasks under the SR on-and SR off-conditions. **(A)** Comparison results of manual dexterity test scores between the SR on- and SR off-conditions. SR (+), stochastic resonance on-condition; SR (–), stochastic resonance off-condition; Blue box, SR on-condition; Pink box, SR off-condition. ^*^P < 0.05. Lines represent the range of the minimum and maximum. Boxes represent the lower, median, and upper quartiles. **(B)** The “visual first” response probability curves of the SR on- and the SR off-conditions in the TOJ tasks. Blue curve, SR on-condition; Pink curve, SR off-condition. **(C)** Delay detection probability curves of the delayed visual feedback detection tasks in the SR on- and off-conditions. Blue curve, SR on-condition; Pink curve, SR off-condition.

Table 2 outlines the measurement results of each index of each block. Figure 2A shows a comparison of the manual dexterity test scores (component score, standard score, and percentile) of the SR on-conditions (a total of six test results of twice each in Blocks 1, 3, and 5) and the SR off-conditions (a total of six test results of twice each in Blocks 2, 4, and 6). The manual dexterity test scores were higher during the SR on-conditions compared with the SR off-conditions (component score, *z* = −2.207, *P* = 0.027, effect size (r) = −2.21; standard score, *z* = −2.214, *P* = 0.027, effect size (r) = −2.21; percentile, *z* = −2.207, P = 0.027, effect size (r) = −2.21; Figure 2A).

**Table 2:** Measurement results of each index of each block.

		**Block-1**		**Block-2**		**Block-3**		**Block-4**		**Block-5**		**Block-6**		**SR (+) Mean**	**SR (–) Mean**
		**SR (+)**		**SR (–)**		**SR (+)**		**SR (–)**		**SR (+)**		**SR (–)**			
		**1**	**2**	**1**	**2**	**1**	**2**	**1**	**2**	**1**	**2**	**1**	**2**		
Manual dexterity test	MD 1 item standard score	14	12	12	12	13	14	13	13	14	12	10	10	13.2	11.7
	MD 2 item standard score	13	13	12	12	12	14	11	13	13	11	11	11	12.7	11.7
	MD 3 item standard score	11	6	1	6	11	11	6	6	11	11	11	11	10.2	6.8
	Component score	38	31	25	30	36	39	30	32	38	34	32	32	36.0	30.2
	Standard score	15	11	8	10	13	15	10	11	15	12	11	11	13.5	10.2
	Percentile rank	95	63	25	50	84	95	50	63	95	75	63	63	84.5	52.3
Temporal order judgment task (sensory bias)	PSE	−1.994		−24.770		–		–		1.802		−7.413		−0.096	−16.092
delayed visual feedback detection task (visuomotor temporal integration)	DDT	233.2		283.4		–		–		205.6		262.4		219.4	272.9
	Steepness	0.041		0.028		–		–		0.057		0.028		0.049	0.028

*SR (+), stochastic resonance on-condition; SR (–), stochastic resonance off-condition; MD 1, manual dexterity test one (placing pegs test); MD 2, manual dexterity test two (threading lace test); MD 3, manual dexterity test three (drawing trail II test); PSE, point of subjective equality; DDT, delay detection threshold: Steepness, steepness of the probability curve for delay detection*.

Figure 2B shows the “visual first” response probability curves of the SR on-conditions (average of two results in Blocks 1 and 5) and the SR off-conditions (average of two results in Blocks 2 and 6). On average, the PSE of the SR off-condition was −16.092 ms, whereas the average PSE of the SR on-condition was −0.096 ms (Table 2; Figure 2B). Therefore, the PSE of the SR on-condition approached 0 ms as compared with the SR off-condition, which showed a reduction of visual dependence (Table 2; Figure 2B).

Figure 2C shows the delay detection probability curves of the SR on-conditions (average of two results in Blocks 1 and 5) and SR off-conditions (average of two results in Blocks 2 and 6). DDT and steepness of the SR on-condition were 219.4 ms and 0.049 on average, respectively, whereas DDT and steepness of the SR off-condition were 272.9 ms and 0.028 on average, respectively (Table 2; Figure 2C). Thus, DDT and steepness of the SR on-condition shortened and increased, respectively, as compared with the SR off-condition, which in turn indicated the improvement of visuo-motor temporal integration during the SR on-condition (Table 2; Figure 2C).

## Discussion

The present results showed that in this one case of DCD, manual dexterity under the SR-on conditions significantly improved immediately, compared with the SR off-conditions. Generally, children with DCD have visual dependence (5–12). In the current case, the PSE of the TOJ task on the day before the experiment was −24.77 ms (Table 1) and the average PSE under the SR off-conditions was −16.092 ms (Table 2; Figure 2B), which indicated visual dependency. However, the average PSE of the TOJ task under the SR on condition was −0.096, indicating a mitigation of visual dependence (Table 2; Figure 2B). Tactile sensation of the hand is a prerequisite for manual dexterity such as object grasping, object manipulation, and handwriting (69–72). Previous studies showed that vibrotactile noise stimulation to the wrist with an intensity of 60% of the sensory threshold improves fingertip tactility and manual dexterity in the affected limbs of patients with stroke (32, 34, 40). Therefore, the improvement of manual dexterity under the SR on-conditions in the current case may have been due to the improvement of tactile sensitivity in the child's hand, which is important for manual dexterity, and the accompanying relief of visual dependency.

In addition, visuo-motor temporal integration is a very important function for manual dexterity (23, 66). In the current case, the DDT and steepness of the delayed visual feedback detection task on the day before the experiment were 275.7 ms and 0.0267, respectively (Table 1), and the average DDT and steepness under the SR off-conditions were 272.9 ms and 0.028, respectively (Table 2; Figure 2C). In contrast, the average DDT and steepness under the SR on-conditions were 219.4 ms and 0.049, respectively (Table 2; Figure 2C). This suggested that the improvement of visuo-motor temporal integration under the SR on-conditions, which, in addition to the reduction of visual dependency, could have contributed to the improvement of manual dexterity following imperceptible vibrotactile noise stimulation in the current case. Therefore, it is possible to hypothesize that the improvement of manual dexterity by SR in the current case was because SR reduced visual dependency and promoted visuo-motor temporal integration.

We did not measure the patient's brain activity; therefore, although the following is completely speculative, the results observed in the current case may have been brought about by the effects of SR on the activity of the central nervous system. Seo et al. (46, 47) demonstrated that imperceptible vibrotactile noise on the wrist increases not only sensorimotor cortex activity but also the activity of the premotor and parietal cortices, which are responsible for tactile sensitivity (46), visuo-motor temporal integration (48), and manual dexterity (24–27). Thus, the positive effects observed in the current case may have been due to activation of the frontal-parietal network in addition to activation of the sensorimotor cortex.

The current case study has several limitations that should be noted. The data could not be analyzed statistically since only a few sensory dependence (TOJ task) and visuo-motor temporal integration (delayed visual feedback detection task) measurements were acquired during the SR on- and off-conditions. Therefore, we cannot conclude that the reason for the significant improvement of manual dexterity under the SR on-conditions shown in the current case was via an improvement of visual dependence and visuo-motor temporal integration. In addition, this was a single case study; thus, future interventional studies with a large sample size are needed to determine the effectiveness of SR for children with DCD. Furthermore, the verification of retention effects after the end of SR administration is also required. In the current study, the SR on- and off-conditions were performed alternately, but the effects obtained under the SR on-condition disappeared under the next off-condition. Therefore, there may be no retention effects after removing SR devices. Thus, future studies designed to investigate retention effects after removing SR devices are also needed. The advantage of the SR phenomenon is that children only wear the devices, the stimulation is below the detection threshold, and children do not need special efforts to use the devices. The combined use of SR with highly effective interventions (73), such as the cognitive orientation to daily occupational performance approach and neuromotor task training, may provide additional benefits to children with DCD.

## Data Availability

The raw data supporting the conclusions of this manuscript will be made available by the authors, without undue reservation, to any qualified researcher.

## Ethics Statement

The experimental procedures were approved by the local ethics committee of the Graduate School and Faculty of Health Sciences at Kio University. There were no foreseeable risks to the case; no personally identifying information was collected. The child and parents provided background information and written informed consent. The procedures complied with the ethical standards of the 1964 Declaration of Helsinki regarding the treatment of human participants in research.

## Author Contributions

SN designed the study, collected and analyzed the data, and wrote the manuscript. MO, TM, SS, and AN provided experimental equipment and evaluation battery and helped with data analyses. AM, EF, and SM supervised the study. All authors read and approved the manuscript.

### Conflict of Interest Statement

The authors declare that the research was conducted in the absence of any commercial or financial relationships that could be construed as a potential conflict of interest.
